# Detection of CTX-M-15 Extended-Spectrum β-Lactamases Producing *Escherichia coli* Isolates from Colostrum and Faeces of Newborn Dairy Calves in China

**DOI:** 10.3390/pathogens10091162

**Published:** 2021-09-09

**Authors:** Zhiyuan He, Sirui Yang, Yulin Ma, Shuyuan Zhang, Zhijun Cao

**Affiliations:** 1 Department of Animal Nutrition and Feed Science, College of Animal Science and Technology, China Agricultural University, Beijing 100193, China; hezhiyuan@cau.edu.cn (Z.H.); 18612777588@163.com (S.Y.); ma18810318038@163.com (Y.M.); s20193040589@cau.edu.cn (S.Z.); 2 State Key Laboratory of Animal Nutrition, Beijing Engineering Technology Research Center of Raw Milk Quality and Safety Control, Department of Animal Nutrition and Feed Science, College of Animal Science and Technology, China Agricultural University, Beijing 100193, China

**Keywords:** dairy calves, colostrum, faeces, ESBL-EC, *bla*_CTX-M-15_ gene, IncHI2 plasmid

## Abstract

Newborn dairy calves are often colonized by multidrug-resistant (MDR) extended-spectrum β-Lactamase producing *Escherichia coli* (ESBL-EC), which pose significant risks to global healthcare. As the first meal of calves, the role of dairy colostrum as a potential source of MDR-*E. coli* has not been well-studied. Here, we report on similar antibiotic resistance patterns of *E. coli* strains, isolated from colostrum fed to dairy calves and their faeces. Four ESBL-EC strains from colostrum and faeces of newborn dairy calves were isolated by double-disc synergy testing and multiplex PCR. Strikingly, isolates from colostrum or faeces were found to have similar MDR profiles, showing a high resistance to cephalosporins and other conventional antibiotics. In addition, coexistence of *bla*_CTX-M-15_ and *bla*_TEM-171_ was detected on a self-transferable plasmid with a typical IncHI2 backbone. To the best of our knowledge, this is the first case reporting on ESBL-EC strains carrying *bla*_CTX-M-15_ and *bla*_TEM-171_ genes, and isolated from faeces and the colostrum stock fed to the dairy calves.

## 1. Introduction

The emergence and dissemination of ESBL-producing *Enterobacteriaceae* of animal origin are rising, and their increased prevalence has become a great challenge to veterinary practitioners around the world. *E. coli* is one of the most common ESBL producers in healthcare settings and often exhibits MDR [1]. As a group of hydrolytic enzymes, ESBL are mainly produced by Gram-negative bacteria conferring resistance to monobactams, penicillins, and cephalosporins.

The most common ESBL, encoded by *Enterobacteriaceae*, belongs to the TEM, CTX-M, SHV, or OXA family. Among them, CTX-M-type β-Lactamase genes (*bla*_CTX-M_) has spread throughout the world since its initial capture from the chromosome of *Kluyvera* spp. to the conjugative plasmids [2]. CTX-M ESBL encoding genes are divided into five clusters, including CTX-M-1, CTX-M-2, CTX-M-8, CTX-M-9, CTX-M-25, based on amino acid sequence identity [3,4]. In the past few years, CTX-M-15 has become one of the most dominant ESBL encoding genes, among humans and food-producing animals globally, suggesting a broad spectrum of reservoirs harboring and spreading these genes [5,6].

The increasing prevalence of highly resistant ESBL-EC, isolated from newborn animals, has become a challenge to clinical therapy. It has been linked to several external factors, including calf housing types, the environment, and postpartum calf management practices [7]. Exposure of newborn calves to antibiotic drugs may also accelerate colonisation and shedding of MDR bacteria [8]. In fact, apart from being an important source of immunoglobulins, nutrients and growth factors [9], dairy colostrum is also the origin of antimicrobial resistance genes (ARGs) or MDR bacteria for newborn calves via feeding practices [10]. Considering the importance of colostrum as a source of MDR bacteria and ARGs, investigations into its potential role as a transmission vector of ESBL-EC are crucial.

In this study, we report on four CTX-M-15 ESBL-EC isolates from faeces and colostrum fed to newborn dairy calves in China. Genotypic and phenotypic identifications of ESBL-producers were undertaken to gain a better insight into partial genomic and plasmid characterisation of the isolates.

## 2. Results

### 2.1. Antibiotic Resistance Profiles of E. coli Strains Isolated from Faeces and Colostrum Fed to Dairy Calves

**Figure 1:**
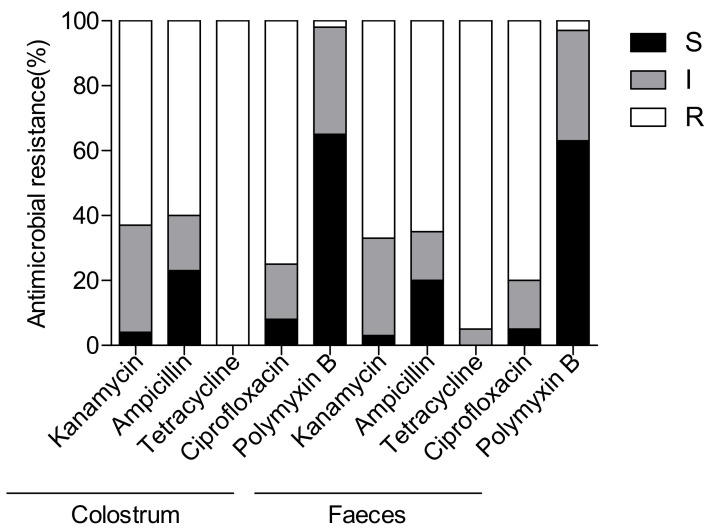
Distribution of antibiotic resistance patterns of *E. coli* strains isolated from faeces and colostrum fed to dairy calves. The proportion of isolates microbiologically resistant to different antimicrobials in the different production systems based on phenotypic characterisation by VITEK method. Five commonly used antimicrobial agents in human clinic and/or animal husbandry are detected in this study. R, resistant; I, intermediate; S, susceptible.

Bacterial strains were isolated from colostrum fed to dairy calves and faecal samples through routine surveillance in Heilongjiang, China, in 2020. A total of 68 *E. coli* isolates were identified using MALDI-TOF MS system and used for further antimicrobial resistance testing trials. As shown in Figure 1, nearly all isolates were found to be tetracycline-resistant, and more than 60% of colostrum or faecal strains were resistant to ampicillin. However, a relatively high rate of susceptibility was detected towards polymyxin B (65.3%; 95% CI: 50.8, 82.1 and 63.7%; 95% CI: 47.3, 78.4, respectively). Importantly, isolates of these two groups were also showing similar resistant rate to other non β-lactams, including kanamycin (62.3%; 95% CI: 45.9, 76.3 and 67.5%; 95% CI: 49.6, 85.3), and ciprofloxacin (74.3%; 95% CI: 58.7, 88.3 and 80%; 95% CI: 63.6, 95.8). These results indicated that antibiotic resistance profiles of *E. coli* strains isolated from faeces and colostrum fed to dairy calves were similar.

### 2.2. Characterisation and Transferability of CTX-M-15 ESBL-EC

**Figure 2:**
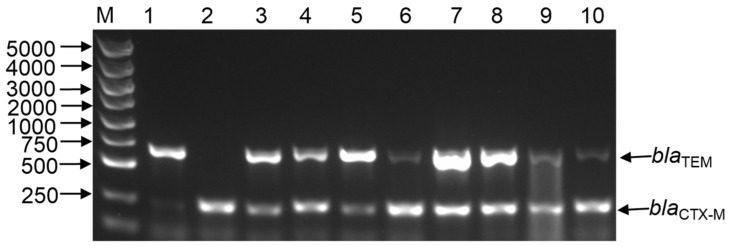
Detection of ESBL genes in *E. coli* isolates. PCR products were separated on 1% agarose gel. M: 100–5000 bp DNA marker; 1: *bla*_TEM_ positive isolate; 2: *bla*_CTX-M_ positive isolate; 3: 1557 isolate; 4: 1584 isolate; 5: 1587 isolate; 6: 1591 isolate; 7: T-1557 isolate; 8: T-1584 isolate; 9: T-1587 isolate; 10: T-1591 isolate.

Based on the high β-lactam resistant rate, we further detected ESBL-EC using phenotypic confirmatory examinations. As a result, four strains (1557 and 1584 from colostrum, 1587 and 1591 from faeces) were recovered from the above *E. coli* strains (Table 1). Antimicrobial resistance testing suggested that 1557 and 1584 were resistant to all antimicrobial agents, but still susceptible to meropenem (Table 2). The presence of *bla*_CTX-M-15_ was also verified by ESBL genotype specific PCR amplification and confirmed by sequencing. As shown in Figure 2, all these four isolates carried *bla*_CTX-M-15_ and *bla*_TEM-171_ genes. Conjugation assays indicated that plasmids of strains 1557, 1584, 1587, and 1591 were transferable to *E. coli* strain J53 at a frequency of 5.58 × 10^−4^, 1.63 × 10^−4^, 1.78 × 10^−4^, and 2.58 × 10^−4^ per donor cell. Interestingly, the transconjugants acquired the major β-lactam resistances of their corresponding donors, but were susceptible to tetracycline, trimethoprim-sulfamethoxazole, meropenem, kanamycin, and ciprofloxacin (Table 2), which implied that the β-lactamase encoding genes and the other resistance genes were on separate replicons. Interestingly, PCR amplification and sequencing demonstrated that *bla*_CTX-M-15_ and *bla*_TEM-171_ genes were also transferred to *E. coli* J5 simultaneously (Figure 2), indicating the possibility of the coexistence of *bla*_CTX-M-15_ and *bla*_TEM-171_ within the same plasmid.

### 2.3. Plasmid Based Replicon Types (PBRT)

**Figure 3:**
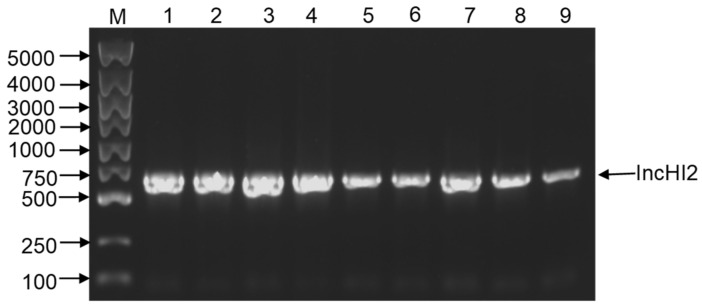
Detection of Inc types of ESBL-producing *E. coli* plasmids according to previous publications of ESBL. PCR products were separated onto 1% agarose gel. M: 100–5000 bp DNA marker; 1: 1557 isolate; 2: 1584 isolate; 3: 1587 isolate; 4: 1591 isolate; 5: T-1557 isolate; 6: T-1584 isolate; 7: T-1587 isolate; 8: T-1591 isolate; 9: IncHI2 positive control isolate.

To further detect the plasmid type mediating the horizontal gene transfer, ESBL-ECs were detected by PBRT assays to obtain specific replicon. As we can see from Figure 3, the plasmids of the above isolates shared a specific IncHI2 iterons, suggesting that the plasmid carrying *bla*_CTX-M-15_ and *bla*_TEM-171_ belongs to the incompatibility group, IncHI2.

## 3. Discussion

Cephalosporins and other antibiotics (aminoglycosides or tetracyclines) have been widely used in dairy farms in China for the treatment of various diseases, including diarrhea, pneumonia, and mastitis [11]. In fact, almost all calves received antimicrobial drug treatment during their first two months, especially in the pre-weaning stage. However, the ESBL-EC isolation rate from food-producing animals has increased, placing continuous pressure on global healthcare. Therefore, the occurrence and dissemination of ESBL-EC isolates with MDR profiles in dairy calves is not unexpected. However, our existing knowledge of the effects of colostrum management on antimicrobial resistance diffusion was limited. Therefore, our present study showed that calves feeding pasteurized colostrum were infected with a markedly large scale of MDR isolates, and more than 60% of all *E. coli* isolates recovered from dairy colostrum or faeces were MDR. In addition, low susceptibility against common β-lactams or the other drugs was observed. This observation was similar to a previous research concerning antimicrobial resistance of *E. coli* in Holstein pre-weaned dairy calves [12], which might be due to the existence of β-lactamase genes and clinically combined antibiotic treatment in dairy farms. Interestingly, elevated MICs of meropenem (0.15 μg/mL) were detected compared with that of recipient *E. coli* J53 (0.03 μg/mL), indicating a possible mutation in one of the chromosomal genes of those *E. coli*. To our knowledge, this was the first report on the detection of CTX-M-15 producing ESBL-EC from colostrum and faeces of dairy calves simultaneously. In fact, infections of those ESBL-EC strains were closely linked to a high incidence rate of calf diarrhea. The cases were even worse in newborn dairy calves due to the limited drug selection and their poor immunity. Indeed, urgent efforts should be taken to reinforce infection control measures. Nowadays, biological treatments are being used to minimize the negative effects of ARGs or bacteria in milk, including heat treatment or ion catalysis [13,14]. Among them, pasteurization is frequently used to decrease microbial contamination and induce antibiotics reduction. However, more effective tools are essential for evaluating the effectiveness of those methods.

In this study, four *E. coli* isolates from calves were detected as resistant to at least five β-lactams. The same phenomena was also found in tetracyclines, fluoroquinolones, aminoglycosides, and carbapenems. Pre-weaned calves were the most susceptible groups to ESBL-EC or the other kinds of MDR bacteria. Indeed, various β-Lactamases resistant genes appeared in lactating cattle were also detected in heifers [15]. Our results showed that *bla*_CTX-M-15_ and *bla*_TEM-171_ genes were detected simultaneously in isolates from faeces of calves and the colostrum fed to calves, which was similar to the detection of CTX-M-15 *E. coli* from cattle or other food-producing animals in East Asia and a national resistance surveillance study of ESBL-EC [5,16]. It was known that, plasmid-mediated horizontal transfer of resistant genes in commensal *E. coli* accelerated the spread of resistance, especially CTX-M-type β-lactamase genes [17,18]. This study detected the existence of the conjugatable plasmid IncHI2 with *bla*_CTX-M-15_ and *bla*_TEM-171_ genes in four ESBL-ECs, which were isolated from faeces of calves, as well as the colostrum that was used to feed calves in the farm. Similar plasmid was previously detected in ST114- *E. cloacae* isolates, mostly carrying *bla*_CTX-M-15_ gene [19]. Importantly, our study observed a relatively high transfer frequency from donors to *E. coli*, indicating the great potential of interspecies transfer of *bla*_CTX-M-15_. Therefore, further surveillance and plasmid characterization were needed to monitor the epidemic spread of such *bla*_CTX-M-15_-carrying IncHI2 plasmids among *Enterobacteriaceae*. Notably, IncHI2 was the most prevalent plasmid type in this study. The existence of complicated resistant gene types could contribute to the large-scale diffusion of plasmids, which mediated better survival in the selective pressure.

It was worth noting that the antimicrobial susceptibility profiles of those strains had been systematically analysed, but relatively small samples limited this research, particularly given the large reserve of heifers in China. In our current study, colostrum samples were collected aseptically to minimize the impacts of environmental contamination and all meconium samples of those calves were also screened for negative ESBL genes to eliminate the influence of maternal factors. Therefore, this study reflected the real impact of colostrum feeding practice and the transmission process from colostrum to faeces on the colonization of ESBL-EC in dairy calves. On this basis, environmental disinfection in the delivery rooms and calf hutches needed to be firmly enforced and revised in pasture. More importantly, control measures of pasteurized colostrum may have an immediate impact by interrupting chains of transmission in newborn dairy calves. Colostrum was the first meal of newborn dairy calves and a vital source of available passive immunity. Our results demonstrated that colostrum was a possible transmission vector of CTX-M-15 ESBL-EC exposure to newborn dairy calves. Therefore, further studies are required to evaluate the effects of feeding colostrum on the prevalence of ESBL-EC and IncHI2 plasmid among dairy farms in China. In summary, this study was an observation experiment on the epidemics of CTX-M-15 ESBL-EC among newborn dairy calves, suggesting the possible spreading chain in communities via colostrum feeding.

## 4. Materials and Methods

### 4.1. E. coli Cells and Reagents

J53 *E. coli* cells were purchased from Transgen company (Beijing, China). Unless otherwise noted, strains were grown in Luria-Bertani broth (LB, Qingdao Hope Bio-technology) or on LB agar plates at 37 °C for 18 h. MacConkey agar quantities were obtained from Luqiao (Beijing, China). Antimicrobial drug standards were purchased from Solarbio (Beijing, China). Kirby-Bauer disks for drug sensitivity test were obtained from Hangzhou Microbial reagent company (Hangzhou, China).

### 4.2. The Farm and Sample Collection

The samples used in this study were collected from a conventional dairy farm, located in the south of Heilongjiang Province in 2020. The farm raised about 3173 heifers and 4945 adult cows of Holstein. It represent the typical dairy production practices of China, where β-lactams are regularly used to treat calf diarrhea, including penicillin and ceftiofur. The sampling protocols were reviewed and approved by the Beijing Association for Science and Technology (ID no. SYXK, 2016-0008). Colostrum samples were randomly collected from 10 healthy Holstein dairy cows 2 h postpartum aseptically, and then pasteurized according to colostrum management method of farm, which were treated at 62 °C for 30 min in 50 mL centrifuge using water bath (HerryTech, Shanghai, China). The samples were stored in the farm freezer until they were used for subsequent bacterial isolation. Touching anus was used to increase meconium excretion, and then faecal samples were randomly collected from 30 individual newborn female calves (0–14 d) with clinical diarrhea from September 2020 to October 2020. All calves were similar in physical conditions, and were fed 4 L of maternal colostrum from certain milking cows during the first 2 h of their lives. Calves were individually arranged in calf hutches to avoid direct contact and bucket fed 4–6 L of raw milk (mixture of raw milk and waste milk) daily during the first month of age. Non-medicated calf starter (25.6% crude protein, 4% crude fat, 24.5% neutral detergent fiber) was offered along with raw milk from day 3 until weaning. Dedicated equipment and sterile gloves were used for rectal fecal sample collection to prevent cross-contamination. No calves received therapeutic antibiotic treatment during the trial.

### 4.3. Bacterial Isolation and Identification

A quantity of 1 mL colostrum sample or 1 g calf faeces was diluted in 9 mL of LB before incubated overnight at 37 °C and then streaked onto MacConkey agar plates using the pure culture law. The suspected colonies with red colour in Maconkey agar plates were confirmed using matrix-assisted laser desorption/ionization time-of-flight mass spectrometry (MALDI-TOF MS) (BrukerDaltonik GmbH, Bremen, Germany). The common genes encoding ESBL were screened by PCR as described previously with minor revisions and confirmed by sequencing [20]. Confirmed isolates were stored in LB containing 30% glycerol at −80 °C for further analysis. All experimental protocols were approved by the institutional ethics committees of China Agricultural University (Approval IDs: XXMB-2012-03-01-1, XXMBB-2012-03-15-1).

### 4.4. Antimicrobial Susceptibility Testing

Antibiotic susceptibility testing was carried out using the VITEK 2 compact system (bioMérieux, Marcy-l’Étoile, France). The minimum inhibitory concentrations (MICs) results of 10 antibiotics (tetracycline, trimethoprim-sulfamethoxazole, amoxicillin-clavulanate, cefepime, aztreonam, meropenem, ampicillin, cefotaxime, kanamycin, ciprofloxacin) were determined using the agar dilution method, according to the breakpoints and interpreted criteria of the Clinical and Laboratory Standards Institute (CLSI) guidelines [21] and the EUCAST guidelines (http://www.eucast.org, accessed on 1 January 2021). Reference strain *E. coli* ATCC 25,922 (Tianhe, Hangzhou, China) was used as the quality control strain.

### 4.5. Phenotypic Screening of ESBL-Producing E. coli

All *E. coli* isolates were screened for the phenotypic identification of ESBL-producers on MacConkey agar containing cefotaxime (2 mg/L), followed by confirmation using double-disc synergy testing, in accordance with CLSI recommendations. Isolates were determined to be positive when the clear zone inhibition of ceftazidime plus clavulanic acid, or cefotaxime plus clavulanic acid, was at least 5 mm larger than their respective single discs [22].

### 4.6. Conjugation Experiment

The horizontal transferability of CTX-M-15 was tested using conjugation assay by broth and filter mating with *E. coli* J53 (azide-resistant) as the recipient strain. The transconjugants were selected on LB agar plates containing 2 mg/L cefotaxime and 100 mg/L sodium azide only after incubating donor and recipient organisms in 4 mL LB for 18 h. The acquired drug resistances of transconjugants were assessed by antimicrobial susceptibility testing. Common ESBL genes in the transconjugants were confirmed by PCR amplification.

### 4.7. Plasmid Based Replicon Types (PBRT)

A Multiplex PCR was adopted to identify specific replicon types of plasmids based on the previously established means [23,24]. PBRT products with similar band profiles were determined to be the same plasmid types.

### 4.8. Statistical Analysis

Statistical analysis was conducted using Graphpad prism 5 (version 5.2.1350.0) and SPASS (version 19.0) software. Data were presented as Mean ± SD. All experimental data were representative of three independent experiments with similar results.

## 5. Conclusions

In conclusion, this study characterised four CTX-M-15 ESBLs-EC strains carrying MDR properties. The study demonstrated the isolation and characterisation of the conjugatable plasmid IncHI2 with *bla*_CTX-M-15_ and *bla*_TEM-171_ genes from faeces of calves and the colostrum that was used to feed calves in the farm. Our findings demonstrate that the plasmid is self-transmissible to other *Enterobacterales,* thereby making them intractable to antibiotics. Further, the study highlights the urgent need to control and monitor the potential spread of such conjugatable plasmid in dairy farms.

## Figures and Tables

**Table 1:** Double Disc Synergy Test (DDST) for confirmation of ESBL-producing *E. coli*.

Clear Zone Inhibition ^1^	Diameter (mm) ^2^			
	1557	1584	1587	1591
CTX	13	11	12	12
CTX/CA	23	19	20	18
CAZ	18	12	12	8
CAZ/CA	25	18	24	15

^1^ Antimicrobial agents are abbreviated as follows: Cefotaxime (CTX), Cefotaxime plus Clavulanic acid (CTX/CA), Ceftazidime (CAZ), Ceftazidime plus Clavulanic acid (CTX/CA). ^2^ The test was considered to be positive when the zone of inhibition of cefotaxime plus clavulanic acid or ceftazidime plus clavulanic acid was >5 mm compared to their respective single discs.

**Table 2:** Antimicrobial susceptibility profiles of different bacterial hosts carrying *bla*_CTX-M_ and *bla*_TEM_-positive plasmids, *E. coli*-J53 transconjugants and the *E. coli*-J53 recipient.

Strains ^1^	MICs (μg/mL) ^2^									
	TET	TES	AMC	CEP	AZT	MPN	AMP	CTX	KAN	CIP
1557	128	8/160	128/64	16	>64	0.15	>256	>256	1024	64
T-1557	1	1/10	128/64	8	16	0.15	>256	>256	0.25	0.25
1584	512	8/320	128/64	16	32	0.15	>256	>256	64	128
T-1584	1	1/10	128/64	8	8	0.15	>256	>256	0.25	0.25
1587	256	8/160	128/64	16	>64	0.15	>256	>256	256	128
T-1587	1	1/10	128/64	8	16	0.15	>256	>256	0.25	0.25
1591	256	8/320	64/32	16	>64	0.15	>256	>256	256	64
T-1591	1	1/10	64/32	8	16	0.15	>256	>256	0.25	0.25
J53	1	1/10	1/0.5	0.25	1	0.03	2	0.25	0.25	0.25

^1^ T-1557, T-1584, T-1587 and T-1591 represent the transconjugations of *E. coli* 1557, *E. coli* 1584, *E. coli* 1587 and *E. coli* 1591. ^2^ MIC, minimum inhibitory concentration. Antimicrobial agents are abbreviated as follows: Tetracycline (TET), Trimethoprim-sulfamethoxazole (TES), Amoxicillin-clavulanate (AMC), Cefepime (CEP), Aztreonam (AZT), Meropenem (MPN), Ampicillin (AMP), Cefotaxime (CTX), Kanamycin (KAN), Ciprofloxacin (CIP). Data are summarised from three different experiments.

## Data Availability

No new data were generated or analysed in support of this research.
